# Uptake of Diagnostic Tests by Livestock Farmers: A Stochastic Game Theory Approach

**DOI:** 10.3389/fvets.2020.00036

**Published:** 2020-02-05

**Authors:** Sibylle Mohr, Rodney Beard, Alasdair J. Nisbet, Stewart T. G. Burgess, Richard Reeve, Matthew Denwood, Thibaud Porphyre, Ruth N. Zadoks, Louise Matthews

**Affiliations:** ^1^Boyd Orr Centre for Population and Ecosystem Health, Institute of Biodiversity, Animal Health and Comparative Medicine, College of Medical, Veterinary and Life Sciences, University of Glasgow, Glasgow, United Kingdom; ^2^Moredun Research Institute, Pentlands Science Park, Midlothian, United Kingdom; ^3^Department of Veterinary and Animal Sciences, Faculty of Health and Medical Sciences, University of Copenhagen, Frederiksberg, Denmark; ^4^The Royal (Dick) School of Veterinary Studies, The Roslin Institute, University of Edinburgh, Midlothian, United Kingdom; ^5^Faculty of Science, Sydney School of Veterinary Science, University of Sydney, Sydney, NSW, Australia

**Keywords:** game theory, sheep scab, disease control, stochastic game, Nash equilibrium, social optimum

## Abstract

Game theory examines strategic decision-making in situations of conflict, cooperation, and coordination. It has become an established tool in economics, psychology and political science, and more recently has been applied to disease control. Used to examine vaccination uptake in human medicine, game theory shows that when vaccination is voluntary some individuals will choose to “free-ride” on the protection provided by others, resulting in insufficient coverage for control of a vaccine-preventable disease. Here, we use game theory to examine farmer uptake of a new diagnostic ELISA test for sheep scab—a highly infectious disease with an estimated cost exceeding £8M per year to the UK industry. The stochastic game models decisions made by neighboring farmers when deciding whether to adopt the newly available test, which can detect subclinical infestation. A key element of the stochastic game framework is that it allows multiple states. Depending on infestation status and test adoption decisions in the previous year, a farm may be at high, medium or low risk of infestation this year—a status which influences the decision the farmer makes and the farmer payoffs. Ultimately, each farmer's decision depends on the costs of using the diagnostic test vs. the benefits of enhanced disease control, which may only accrue in the longer term. The extent to which a farmer values short-term over long-term benefits reflects external factors such as inflation or individual characteristics such as patience. Our results show that when using realistic parameters and with a test cost around 50% more than the current clinical diagnosis, the test will be adopted in the high-risk state, but not in the low-risk state. For the medium risk state, test adoption will depend on whether the farmer takes a long-term or short-term view. We show that these outcomes are relatively robust to change in test costs and, moreover, that whilst the farmers adopting the test would not expect to see large gains in profitability, substantial reduction in sheep scab (and associated welfare implications) could be achieved in a cost-neutral way to the industry.

## Introduction

Effective policies for livestock disease control and surveillance ideally require that we take into account individual decision-making and behavior. Even so, new diagnostic tests and control measures are typically developed without an assessment of whether they are likely to be taken up by the farming community. An example of current importance is the potential uptake of a new diagnostic test for sheep scab (Psoroptic mange), which is one of the most important diseases in terms of welfare and economic impacts for sheep farmers in the United Kingdom (UK) (1).

Sheep scab is a highly contagious disease, caused by infestation with an ectoparasitic mite (*Psoroptes ovis)*, prompting an allergic reaction and intense irritation to the animals resulting in rubbing and scratching behavior that leads to large and painful skin lesions (2, 3). Psoroptic mange is not only a major animal welfare concern but also imposes a significant economic burden on livestock industries in many locations worldwide (1). In the UK, incidences of sheep scab dramatically increased following the deregulation of compulsory sheep scab preventative treatment in 1992, with the number of outbreaks rising from just under 100 per year to an estimated 7,000 in 2003 and 2004 (4–6). Since then, infestation by the parasite has been made notifiable in many countries and, in Scotland, the Scottish Government has collaborated with industry through the Scottish Sheep Scab Initiative to enable control of the disease since 2004.

Transmission of sheep scab typically occurs through direct contact with an infested animal or by contact with contaminated fomites in an infested environment, for instance with fence posts, farm machinery, or contaminated wool. Importantly, continuous incursions of infestation happen between neighboring farms (7, 8), particularly when these farms keep sheep in adjacent fields with shared rubbing areas or when there are gaps in common fence-lines. Because of this, it is typically recommended that neighbors should treat at the same time to achieve maximum effect and protection. Currently, sheep scab can only be diagnosed at the late clinical stage, meaning that infestation is able to spread between animals and between farms prior to detection and treatment. At present, farmers use various chemicals to treat the sheep, including organophosphate plunge dipping or injection with macrocyclic lactones (ML) (3). This has the potential to be detrimental to the environment and, crucially, it has been shown that mites have evolved to become resistant to ML chemicals (9, 10). It is well-established that clinical infestation with sheep scab substantially reduces growth and productivity and, if left untreated, can even kill.

If farmers were able to diagnose and effectively treat sheep strategically, at an early stage of infestation, this would not only benefit the health and welfare of the sheep, but it would also avoid farmers' financial losses from rearing poorly performing animals, as well as reducing transmission to neighboring flocks. One new control option is a recently developed diagnostic blood test (an ELISA test), which can detect sheep scab infestation in sub-clinically infested animals (11, 12). This ELISA test employs a single recombinant antigen, and importantly, is capable of accurately detecting *P. ovis* infestation in sheep at the subclinical stage (12). Such a test would allow the infestation to be identified before the advent of clinical signs, reducing the risk of developing clinical disease and also limiting spread.

The question of whether individuals are likely to adopt an intervention can be studied using game theory. Game theory is a mathematical approach to decision making which captures at its core the idea of strategic interactions, where “strategic” refers to the fact that the decision made by one individual is influenced by the decisions made by others, with classic examples being bargaining or bluffing in cards games. Game theory is such a powerful tool that it has been used to examine a wide range of strategic interactions in social, economic and biological systems, such as conflicts over fishing rights, weapons arms races, pricing strategies among competing firms, and the uptake of interventions in human medicine (13–17). For example, application to the uptake of vaccines in human medicine has shown that if there is any risk or cost associated with vaccination then individual self-interest can prevent eradication of a vaccine-preventable disease (14).

The origins of game theory are typically attributed to the mathematical proof of the minimax theorem by von Neumann in 1928, which established what was later called *Nash equilibrium* for strictly competitive games (18, 19). In general, game theory describes strategic interactions of two or more rational decision makers (or players), where each individual's decision (or actions) jointly determine an outcome that affects them all.

**Figure 1:**
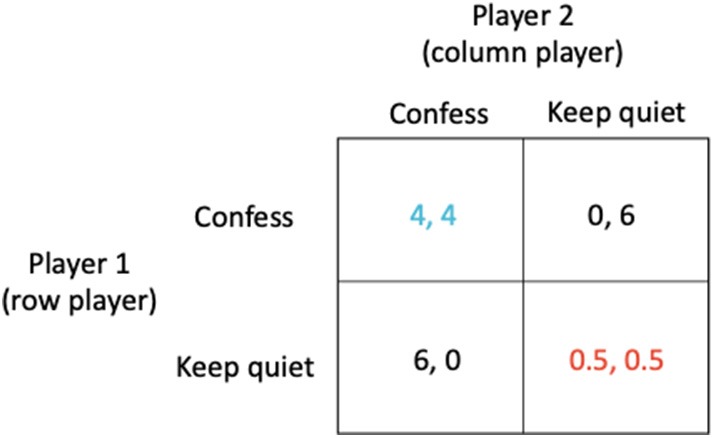
The *prisoner's dilemma* in which (i) if prisoner A confesses while prisoner B does not, the one who confesses will be released immediately (0 year sentence) while the other receives a 6 year sentence, (ii) if neither confesses, each receives just a 0.5 year sentence, and (iii) if both confess, they each receive a 4 year sentence.

The most prominent and well-known example for a simple strategic game is what is known as the *prisoner's dilemma* (20). Two prisoners (A and B) are accused of a crime, for instance robbing a bank together. They are kept separate by the police and are individually presented with a bargain. If prisoner A confesses while prisoner B does not, the one who confesses will be released immediately and the other will spend 6 years in prison. If neither confesses, each will be imprisoned for just 0.5 years; this outcome which has the lowest combined sentence for both players is known as the *social optimum* (shown in red in Figure 1). If both confess, they will each be jailed 4 years. Crucial to determining the outcome is the observation that although neither prisoner knows whether the other has confessed, each prisoner knows that whatever the behavior of the other, they can improve their outcome by confessing (see Figure 1).

The outcome arrived at when each prisoner acts in their own self-interest is known as the *Nash equilibrium* (shown in blue in Figure 1). However, in these circumstances, when each prisoner acts in their own self-interest, both end up worse off (a 4 year sentence) than if they had acted in accordance with the best solution for all (the *social optimum* which corresponds to a sentence of just 0.5 year).

In the context of veterinary disease control, relatively little research has applied game-theoretic techniques, mostly using the standard static strategic-form game approach (21, 22). Here, we combine epidemiological and economic parameters in type of game called a *stochastic* game that aims to analyse the adoption of the new diagnostic ELISA test for sheep scab in Scotland, where sheep scab is a notifiable disease requiring treatment upon confirmatory diagnosis.

Stochastic games extend traditional strategic-form games (18, 23), such that they are responsive to dynamic situations where the environment changes in response to players' choices. Stochastic games were first introduced by Shapley in 1953 who established the idea of multiple states, and at each stage of the play the players chose an action in the game dependent on the current state (24, 25). The set actions (or *strategies*) that each player decides on, together with the current state, determine not only the stage *payoff* that each player receives but also the probability distribution governing the transitions between states. Thus, stochastic game theory provides a suitable mathematical framework for assessing if, and under which circumstances, farmers are likely to adopt the new diagnostic test for subclinical sheep scab by providing a mathematical framework which enables us to capture different risk states inherent to epidemiological problems and probabilistic transitions between these states.

Our aim is to use a stochastic game to answer the question whether farmers will use the newly available diagnostic test and treat early or whether they will wait and treat on clinical diagnosis only. Here, the term stochastic means that we are analyzing a game with different states of *infestation risk*s—states of high, medium, or low risk of infestation. The current state depends on the previous state and the test adoption decision by the farmers. Because sheep scab can spread between neighboring flocks, the decision a farmer's neighbor makes affects their risk of infestation. Therefore, whether a farmer believes his flock might be infested and should be tested will depend to some extent on the decisions his neighbor takes. If a neighboring farm had sheep scab last season the farmer's flock might be at high risk of being infected this year, whereas if his neighbor was free of infestation, the farmer might consider his flock to be at low risk of being infected this year. Moreover, in this situation, strategic interactions arise because the farmer may consider his animals at low risk if his neighbor controls infestation by using the diagnostic test. He may “free-ride” on the protection afforded by his neighbor and choose not to adopt the test. Such outcomes can be suboptimal for disease control in the population as a whole.

The paper is organized as follows: First we introduce the basic assumptions underpinning our sheep scab model. We then introduce the basic game-theoretic concepts and definitions for a simple strategic game. This we then extend to a stochastic-game set-up, which we illustrate with a simple example. Finally, we present our multi-state sheep scab test-adoption game, along with our findings in terms of economic and epidemiological implications as well as a discussion of the limitations of the current approach.

## Characterization of the Game

Before describing the stochastic game in mathematical terms, we introduce our underlying assumptions: The stochastic game presented here is designed to capture the decisions made by two neighboring farmers when confronted with the choice of either adopting the diagnostic test for subclinical sheep scab or not. We assume that a farmer believes his flock to be (i) at high risk of infestation in the current year, if either he or his neighbor suffered clinical sheep scab in the previous year, (ii) at low risk of infestation if both farms were free of infestation last year, (iii) and at a medium risk of infestation if sheep scab was diagnosed using the new test and then treated at the subclinical stage.

The basic game-theoretic concepts are *players* (the decision makers, i.e., the farmers), *strategies* (alternatives among which each player chooses), and *payoffs* (such as financial gains) among the possible outcomes of the game. Fundamentally it is assumed (i) that all players have consistent preferences and behave rationally in the sense of consistently choosing an option that maximizes their individual payoffs based on their beliefs and knowledge at the time of decision-making and (ii) that the specification of the game and the players' payoffs and rationality are common knowledge among the players.

There are multiple factors that may influence whether farmers adopt a new test, such as (i) the cost of the test, (ii) the expected cost of the disease, (iii) and the cost of treating sheep scab. We assume that the current treatment protocol is that sheep are treated when clinical signs are observed, which involves physical examination of individual sheep, locating lesions, followed by diagnosis through skin-scraping by a veterinary surgeon.

The financial profits (*payoffs)* made by a farmer depend on the revenue from his sheep, which will be reduced if they become diseased, together with the costs of testing and treating the animals. At the time when the farmer decides whether to adopt the new diagnostic test, he does not know whether his flock is infected. Hence, he will have to make his decision based on the payoff he *expects*, which will depend on whether he considers his flock at *high, medium* or *low risk* of infestation. The problem can be presented as a stochastic game matrix, with a *high, medium*, and *low risk* state and farmer *payoffs* that depend on the state and their chosen actions (to adopt the test or not to adopt the test). Key to the decisions made by the farmers is also how they weigh up the immediate costs and benefits of adopting the test, which may not pay off in the current year if test costs are high, vs. the long-term benefits of moving to a lower risk state, should both farmers adopt the test. The extent to which a farmer values the short term vs. the long term can be captured by including a *discount factor*. The discount factor captures the farmer's preference for a reward now vs. a future reward (also known as a time preference). Game theory then tells us the decision they arrived at when individuals act according to rational self-interest, known as the *Nash equilibrium*, and also whether this represents the best outcome for both farmers, and we refer to the outcome with the highest combined payoffs for both farmers as the *social optimum*. In the case of the stochastic game, the solution (*Nash equilibrium* or *social optimum*) not only specifies the decision opted for but also determines the relative amount of *time* spent in each state over the long run.

## Notations, basic Definitions, and Stochastic Games

### Basic Definitions

Throughout the paper we apply standard terminology and notation from classical game theory. Before defining how to solve a stochastic game it is useful to illustrate how to solve a standard static game: A simple two-player game is defined by a matrix pair (A, B), specifying the *payoffs* for the row player (player 1) and the column player (player 2), respectively. It is assumed that all game matrices A and B are *n* by *n*, corresponding to a set of *n* strategies available to the players.

For example, the *prisoner's dilemma* game described above (Figure 1) has two strategies available to the players which are “Confess” or “Keep quiet.” Formally, the game is represented by the matrices A (for the row player) and B (for the column player)

$$\begin{array}{l} A=\;\left(\begin{array}{cc} 4 & 0\\ 6 & 0.5 \end{array}\right)\;B=\;\left(\begin{array}{cc} 4 & 6\\ 0 & 0.5 \end{array}\right)\; \end{array}$$

The decisions made by each player are represented by vectors of probabilities over the available strategies. In this case, the strategy vectors (1, 0) and $\left(\begin{array}{c} 1\\ 0 \end{array}\right)$ for the row and column players, respectively, represent the Nash equilibrium.

The strategies “Confess” or “Keep quiet” are examples of what are known as *pure strategies*. In general, a player's strategy may be a probability distribution over the available options, known as a *mixed strategy*, which in the above case could be represented by the strategy vectors (*x*, 1 − *x*) and $\left(\begin{array}{c} y\\ 1-y \end{array}\right)$. Here, the strategy vector (1,0) indicates that the row player will play the first pure strategy “Confess” with probability 1 and the second pure strategy “Keep quiet” with probability 0. Similarly, the strategy vector $\left(\begin{array}{c} 1\\ 0 \end{array}\right)$ indicates that the column player will play the first pure strategy “Confess” with probability 1 and the second pure strategy “Keep quiet” with probability 0. Thus, the Nash equilibrium is both players confessing (Figure 1, blue payoffs), with payoff of 4 for each player. The strategy vectors (0, 1) and $\left(\begin{array}{c} 0\\ 1 \end{array}\right)$ represent the *social optimum* (both players keeping quiet, Figure 1, red payoffs).

Returning to the general case of *n* available strategies, if the row player chooses the strategy *x* and the column player chooses the strategy *y*, player 1 receives payoff A(*x,y*) and player 2 receives B(*x,y*). The strategy vectors X^*^ and Y^*^ represent a Nash equilibrium when

(1) $$\begin{array}{l} {\left(AY^{*}\right)}_{i}\leq X^{*}AY^{*}=v^{1}\; \end{array}$$

(2) $$\begin{array}{l} \text{and }{\left(X^{*}B\right)}_{i}\leq X^{*}BY^{*}=v^{2}\; \end{array}$$

where *v*^1^ is the value to player 1 and *v*^2^ the value to player 2 at the Nash equilibrium. The term ${\left(AY^{*}\right)}_{i}$ is the *i*th element of AY^*^, giving the payoff to player 1 playing the *i*th action against player 2 playing Y^*^. Similarly ${\left(X^{*}B\right)}_{i}$ is the payoff to player 2 playing the *i*th action against player 1 playing X^*^.

The inequalities state that the value to player 1 playing a pure strategy against player 2 playing their Nash equilibrium strategy is always less than or equal to player 1's optimal value, *v*^1^ (and vice versa). This is because player 1 will not do better than earn *v*^1^ against player 2 playing Y^*^ by definition of the Nash equilibrium (and vice versa). Therefore, these inequalities must be satisfied by a Nash equilibrium and hence are a necessary condition for a Nash equilibrium.

To exclude the possibility that strategies other than a Nash equilibrium might satisfy the inequalities, we also show that the inequalities are *sufficient* i.e., that *if* they are satisfied, they represent a Nash equilibrium. To demonstrate this, we consider any old strategy X = (*x*_1_, *x*_2_, *x*_3_, *x*_4_, .., *x*_*n*_) where ∑*x*_*i*_ = 1 and multiply Equation (1) by each *x*_*i*_ and sum the equations to obtain

(3a) $$\begin{array}{l} {\sum_{i=1}^{n}x_{i}\left(AY^{*}\right)}_{i}\leq \sum_{i=1}^{n}x_{i}\;X^{*}AY^{*} \end{array}$$

(3b) $$\begin{array}{l} i.e.,\\ \;{\sum_{i=1}^{n}x_{i}\left(AY^{*}\right)}_{i}\leq {\;X}^{*}AY^{*} \end{array}$$

(3c) $$\begin{array}{l} i.e.,\\ \;XAY^{*}\leq X^{*}AY^{*} \end{array}$$

Consequently, the value to player 1 when playing X^*^ is always greater than or equal to the associated with any old strategy *X*. This demonstrates that X^*^ is the best response to Y^*^. A similar line of reasoning [multiplying Equation (2) by *y*_*i*_, the elements of Y where Y is any old strategy adopted by player 2] demonstrates that Y^*^ is the best response to X^*^. Therefore, the inequalities 1 and 2 are a necessary and sufficient condition, meaning that (X^*^, Y^*^) is a Nash equilibrium if and only if the inequalities are satisfied.

### The Stochastic Game

#### Overview and Example

A stochastic game differs from a static game (outlined above) in three respects: (i) payoffs are specified for multiple states of the system, (ii) transition probabilities between states need to be specified, (iii) the value to a player depends not just on the payoff from the current state but on the discounted sum of payoffs from future states visited. This third component requires an additional parameter, the *beta discounted reward parameter* (β) (here also referred to as *discount factor*), which can take on values between 0 and 1. This parameter is the weight given to next year's payoff relative to the current payoff. Taking the extreme cases, if β = 0, next year's payoff carries no weight in the decision making. If β = 1, next year's payoff carries equal weight to the current payoff (see the Appendix for further details).

**Figure 2:**
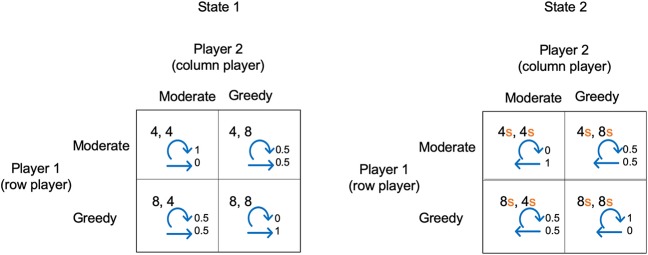
A simple stochastic game for resource competition. The players' pure strategy set is to be “Moderate” or “Greedy.” For *s* < 1, state 2 is the low resource state and state 1 the high resource state. Each quadrant contains the payoffs to each player, plus annotated arrows. The straight arrows are annotated with the probability of moving to the alternative state and the curved arrows are annotated with the probability of remaining in the current state.

The example illustrated in Figure 2 shows a 2-state stochastic game. The diagram shows the payoffs for each player in each state and for each pair of player choices. In this example, for simplicity, the payoffs in state 2 are proportional to the payoffs in state 1 (determined by a scaling factor s). Figure 2 also shows the transition probabilities. For example, the top left box for state 1 shows that if each player makes choice 1, they will each receive a payoff of 4, and given these choices, the probability of remaining in state 1 is 1 and the probability of transitioning to state 2 is 0.

We have chosen a simple example so that in either state choice 1 always gives a lower payoff than choice 2. We can view choice 1 and choice 2 as being “Moderate” or “Greedy” in the case of competition for resources. For s <1, state 1 is the state with high resource levels and state 2 with low resource levels. Both players being “Moderate” in state 1 results in remaining in state 1, whereas if both players are “Greedy” there is a transition to state 2. Conversely, both players being “Moderate” in state 2 means a transition to state 1, whereas both being “Greedy” means remaining in state 2. Whenever one player is “Moderate” and the other “Greedy” there is a 0.5 probability of transitioning to the other state.

**Figure 3:**
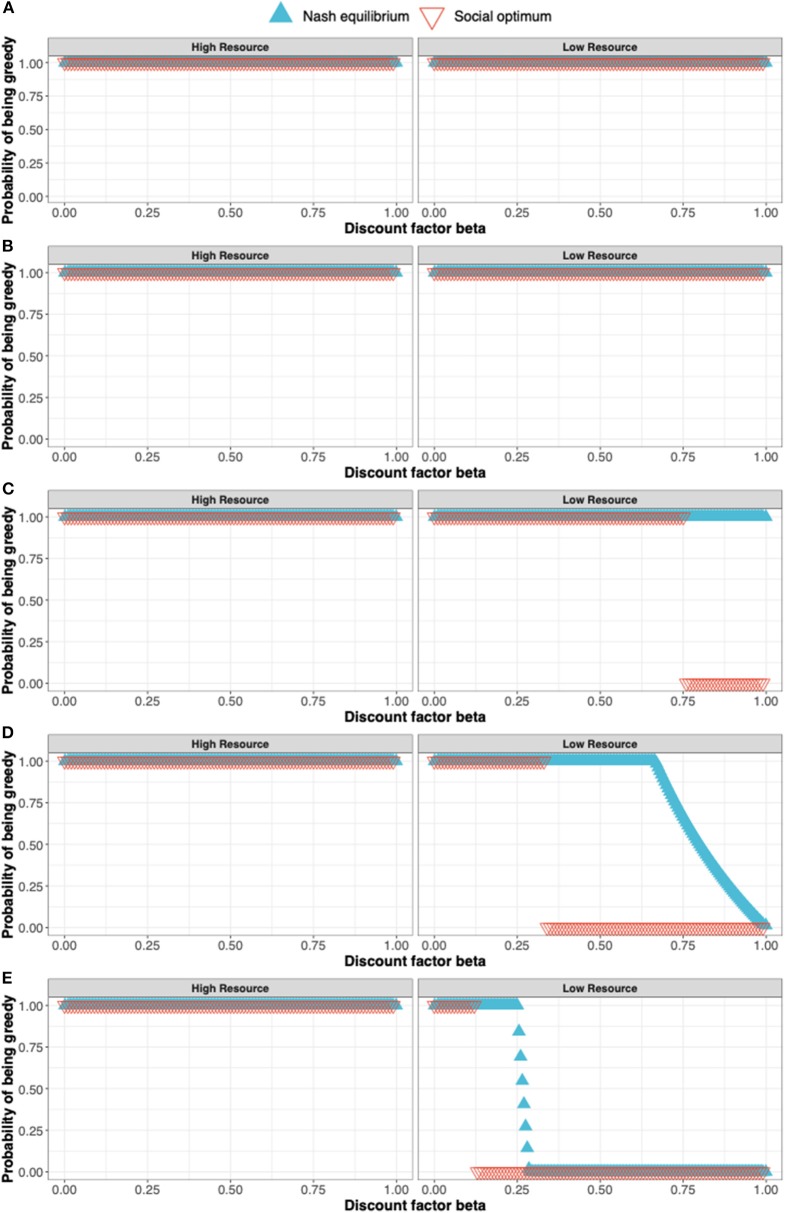
The solution to a simple stochastic game representing resource competition showing the Nash equilibrium (blue triangles) and social optimum (red triangles). The states of the stochastic game are a high resource and low resource state and the pure strategies available to the players are to in either state to either be a Greedy or Moderate user of the resource. The solutions shown in **(A–E)** correspond to *s* = 1.0, 0.8, 0.6, 0.4, and 0.2 where s is the parameter determining the payoff in the state 2 (the low resource state) relative to the payoff in the high resource state.

The procedure for determining the Nash equilibrium and social optimum for a stochastic game such as this is outlined in the Appendix. Here, we illustrate the concepts by discussing the solution for *s* = 1.0, 0.8, 0.6, 0.4, and 0.2 (see Figures 3A–E).

- When *s* =1.0 (Figure 3A), the Nash equilibrium and social optimum for both players is to be “Greedy” in either state. The long-term equilibrium is for the players to be in state 2, both earning the maximum reward of 8 at each time point.
- When *s* = 0.8 (Figure 3B), payoffs in state 2 are 0.8 those of state 1 but the Nash equilibrium and social optimum remain at “Greedy” for both players in each state.
- When *s* = 0.6 (Figure 3C), the social optimum shifts in state 2 from being “Greedy” to being “Moderate” for high values of the discount factor β i.e., the players can achieve greater payoffs by being “Moderate” in state 2 when β is large. For ease, consider the case β = *1*. Under the Nash equilibrium of being “Greedy,” in the long-run both players remain in state 2 earning a payoff of 8s (=4.8). Under the social optimum of both being “Moderate,” the players flip between a payoff of 8 and 4s, giving an average payoff of (8 + 4s)/2 (= 5.2). The social optimum is not a Nash equilibrium as shown by the following argument: If one player were to defect from the social optimum and be “Greedy” in state 2, their payoff would increase to 8s in state 2. Under this scenario, both players spend 1/3 their time in state 1 and 2/3 in state 2. Thus, the long-term payoff for the defecting player would be 8(1 + 2s)/3. This exceeds their payoff at the social optimum (8 + 4s)/2 provided *s* > 0.4. Since a player can increase their payoff by defecting from the social optimum, it cannot be a Nash equilibrium.
- When *s* = 0.4 (Figure 3D), we see a change in the Nash equilibrium with the Nash equilibrium now coinciding with the social optimum for β = 1.
- When *s* = 0.2 (Figure 3E), the Nash equilibrium and social optimum continue to coincide when β =1.

## The Sheep Scab Test Adoption Game: A Multistate Model Set-Up

For the stochastic game we assume voluntary test adoption of the new diagnostic ELISA test under a given perfect test regime, assuming a perfect test sensitivity and test specificity [see Supplementary Material for implementation of a multi-state setup under an imperfect test regime (26)]. Based on infestation status and test adoption decisions taken the previous year, a farm may be at high, medium, or low risk of infestation this year. In the model, last year's decisions and infestation status determine the decisions the farmer takes this year, and along with the resulting farmer payoffs.

To determine the payoffs, we used epidemiological parameters and estimated costs associated with sheep scab prevalence, which were derived from the literature (2, 5, 11) and are summarized in Tables 1, 2. Traditional treatment costs were obtained for two different treatments, organophosphate plunge dipping and an injectable formulation using a macrocyclic lactone. Subsequent results are presented for the slightly cheaper option of dipping.

**Table 1:** Sheep scab economic costs and epidemiological parameters derived from literature.

**Parameter**	**Description**	**Default value**	**Source**
C*_*Diag*_*	Cost per head of clinical diagnosis (£100 per flock)	0.21	ADAS (2)
*C_*Test*_*	Cost per head of new subclinical test (£160 per flock)	0.33	
C*_*Treat*_* (dipping)	Cost per head of dipping	0.61	ADAS (2)
C_Treat_ (injected)	Cost per head of injecting	0.65	ADAS (2)
Subclinical*_*severity*_*	Fraction of revenue lost in subclinical infestation relative to clinical infestation	0.15	ADAS (2)
Disease*_*cost*_*	Disease cost per clinical infestation	6.54	ADAS (2)
*ϕ_1_*	Proportion of flock becoming infested (subclinical or clinical)	132/160	Burgess et al. (11)
*ϕ_2_*	Proportion of flock progressing to clinical infestation	27/160	Burgess et al. (11)

**Table 2:** Assumed or derived parameters.

**Parameter**	**Description**	**Default value**
C*_*Test*_*	Cost of new subclinical test	1.6^*^C*_*Diag*_*
*R_*H*_*	Revenue from a healthy animal	20
*R_*C*_*	Revenue from clinical animals	*R_*H*_* – Disease*_*Cost*_*
*R_*S*_*	Revenue from a subclinical animal	*R_*H*_*– Subclinical*_*Severity*_* Disease*_*Cost*_*
*R_*TT*_*	Revenue from treated animal	0.8 R*_*H*_*+ 0.2R*_*S*_*
*θ_*L*_*	Probability of flock being infested in the low risk state	0.0138 [fitted to Rose et al. (5)]
*θ_*H*_*	Probability of flock being infested in the high risk state	0.585 [fitted to Rose et al. (5)]
*θ_*M*_*	Probability of flock being infested in the medium risk state	0.5(*θ_*L*_* + *θ_*H*_*)

The cost associated with the traditional approach of diagnosing at the subclinical stage is the vet's call-out fee (assumed to be £100 per flock). The cost associated with using the new subclinical diagnostic test is assumed to be the vet's call-out fee plus an additional £60 per flock (27). Costs per head in Table 1 are derived from reported flock costs (2). Thus, the new test is 1.6 × more expensive than the *status quo* clinical diagnosis cost.

Note that the values for the stage payoffs are determined by what the farmer perceives his risk of infestation to be, which may not be an accurate reflection of reality. We considered four scenarios for the test-adoption game:

1) The farmer's flock is **uninfested** and he **does not** adopt the new ELISA test with payoff
2) The farmer's flock is **infested** and he **does not** adopt the test.
3) The farmer's flock is **uninfested** and he **does** adopt the new ELISA test.
4) The farmer's flock is **infested** and he **does** adopt the new ELISA test.

### Expected Payoffs

Under scenario 1, the payoff *P*_1_is represented by *P*_1_ = *R*_*H*._

Under scenario 2, a proportion of the farmer's flock *(*φ_1_ – φ_2_*)* will be subclinically infested with revenue *R*_*S*_, of which proportion φ_2_progresses to clinical infestation with revenue *R*_*C*_. At this point infestation will be identified and the whole flock will be treated at cost *C*_*Treat*_. Therefore, under scenario 2, the farmer's payoff, *P*_2_, would be

$$\begin{array}{l} P_{2}=\;\left(1\;-\varphi_{1}\right)R_{H}+\;\left(\varphi_{1}-\varphi_{2}\right)R_{S}+\varphi_{2}R_{C}-\left(C_{Diag}+C_{Treat}\right) \end{array}$$

Under scenario three, the test costs *C*_*Test*_ are included for the calculation of the stage pay-off *P*_3_,where

$$\begin{array}{l} P_{3}=\;R_{H}-C_{Test} \end{array}$$

Under scenario 4, using the diagnostic test and treating infested animals prevents the flock from progressing to the clinical state. Assuming revenue from flock *R*_*H*_ and test and treatment costs *C*_*Test*_ and *C*_*Treat*_, the stage payoff, *P*_4_ will be

$$\begin{array}{l} P_{4}=\left(1-\varphi_{1}\right)R_{H}+\varphi_{1}R_{TT}-\left(C_{Test}+C_{Treat}\right). \end{array}$$

#### Calculating Expected Payoffs

Another consideration is that because the farmer does know whether his flock is infested or not, he derives his decision to adopt the test or not from what he *anticipates* his payoff to be. Here, we assume that the farmer is risk neutral and therefore that expected payoffs are a linear combination of the payoffs for an infected and uninfected flock. For instance, suppose he thought there was 30% chance (probability of 0.3) that his flock is infected (i.e., a 70% chance or 0.7 probability that it is uninfested). If he decides to adopt the test, his anticipated (or expected) pay-off would be a weighted average of that under scenarios 3 and 4 i.e., the expected pay-off would be 0.7*P*_3_ + 0.3*P*_4_. In general, if the farmer thinks his flock has a probability θ of being infested and he does adopt the test, his expected pay-off is

$$\begin{array}{l} \left(\text{1}-\theta \right)P_{3}+\theta P_{4} \end{array}$$

Conversely, if the farmer thinks his flock has a probability θ of being infested, and he does not adopt the test the expected pay-off is

$$\begin{array}{l} \left(\text{1}-\theta \right)P_{1}+\theta P_{2}. \end{array}$$

### Risk of Infestation and Payoff Matrices

We distinguish the 3 states by the probability θ_*K*_ of the flock being infested in each state with state probabilities θ_*K*_={θ_*L*_, θ_*M*_, θ_*H*_*}* denoting the low, medium and high risk states, respectively.

The payoff matrices stating the expected payoffs (the weighted mean across the uninfested and infested scenarios) for a given action in a given state (θ_*L*_, θ_*M*_, θ_*H*_) are defined in Table 3.

**Table 3:** Payoff matrix stating the expected payoffs for a chosen action in a given state (θ_*L*_, θ_*M*_, θ_*H*_), where θ_*K*_ represents the probability of the flock being infected in low, medium, and high risk states, respectively (θ_*K*_= {θ_*L*_, θ_*M*_, θ_*H*_}).

		**Farmer 2**	
	**Risk state**	**Does not adopt**	**Adopts**
Farmer 1	Does not adopt	*(1 – θ_*K*_)*P_1_ + *θ_*K*_*P_2_, (1 – *θ_*K*_*)P_1_ + *θ_*K*_*P_2_	*(1 – θ_*K*_*)P_1_ + *θ_*K*_*P_2_, (1 – *θ_*K*_*)P_3_ + *θ_*K*_*P_4_
	Adopts	*(1 – θ_*K*_*)P_3_ + *θ_*K*_* P_4_, (1 – *θ_*K*_*)P_1_ + *θ_*K*_* P_2_	*(1 – θ_*K*_*)P_3_ + *θ_*K*_* P_4_, (1 – *θ_*K*_*)P_3_ + *θ_*K*_* P_4_

The infestation probabilities, θ_*L*_ and θ_*H*_ were estimated by fitting a Markov chain (see Supplementary Material) to 10 years of historical sheep scab outbreak data in the UK, comprising approximately 400 farms in total (5). The data available for these farms was their current infection status and the number of outbreaks in the previous 10 years [illustrated in Figure 1 of (5)]. A Markov chain was simulated for a pair of farms in which the probability of infestation is low (θ_*L*_) if neither were infected in the previous year, or high (θ_*H*_) if one or both were infested in the previous year. Maximum likelihood was used to determine the values of θ_*H*_ and θ_*L*_ that provided the best fit to the observed distribution of outbreaks. This model fitting therefore exploits the temporal autocorrelation in these data. No information on θ_*M*_^1^ could be obtained as the subclinical test was not in use. Thus, θ_*M*_ was set equal to the mean of θ_*H*_ and θ_*L*_.

### Outcome Probabilities

In order to decide whether the farmers will consider themselves in a high, medium, or low risk state next season depends on the possible outcomes for this season which in turn depend on the farmers' actions. At the end of the season, the four possible outcomes (allowing for an imperfect test) for a flock are:

(1) clinical infestation is observed and treated,
(2) subclinical infestation is correctly identified with the new test and treated,
(3) subclinical infestation is incorrectly identified with the new test and treated, and
(4) the absence of infestation is correctly identified.

Outcome 1 (clinical infestation is observed and treated) will occur if a flock is infected and also progressed to clinical infestation without the farmer having tested the flock. Outcome 2 (subclinical infestation is correctly identified and treated) will happen if the flock was infected and tested positive. Outcome 3 (subclinical infestation is incorrectly identified) will occur if the flock was uninfected and testing results in a false positive. Outcome 4 (no infestation is observed) will happen if the flock is uninfected and testing returns no false positives.

Given the probability θ of a flock being infected, A_1_(θ), A_2_(θ), A_3_(θ), and A_4_(θ) represent the probabilities of outcomes 1, 2, 3, and 4 if the farmer adopts the test. Similarly, DA_1_(θ), DA_2_(θ), DA_3_(θ), and DA_4_(θ) represent the probabilities of outcomes 1, 2, 3, and 4 if the farmer does not adopt the test.

Here, we specify the vectors A(θ) and DA(θ) for the case of a perfect test. If the farmer *does not adopt* the test, infestation can only be identified at the clinical stage. Thus, the only possible outcomes are 1 (clinical infestation) and 4 (no infestation) which occur with probabilities θ and *1*–θ. If the farmer *does adopt* the test, the only possible outcomes are 2 (subclinical infestation identified) and 4 (no infestation) which again occur with probabilities θ and *1*–θ, respectively. Thus, the vectors of outcome probabilities for adopting the test *A*(θ) and not adopting the test *DA*(θ) are

$$\begin{array}{l} A\left(\theta \right)=\;\left(\begin{array}{c} 0\\ \theta \\ 0\\ 1-\theta \end{array}\right),DA\left(\theta \right)=\;\left(\begin{array}{c} \theta \\ 0\\ 0\\ 1-\theta \\ \; \end{array}\right). \end{array}$$

### Transition Probabilities Between High, Medium, and Low Risk States

The outcomes described above determine whether a farmer is in a high, medium, or low risk state *next* season. In other words, the outcomes determine the probabilities of transitioning to the high, medium or low risk state. We assume that **H, M**, and **L** are 4 × 4 matrices, which satisfy *L*_ij_ + *M*_*ij*_ + *H*_ij_ =1. ***L***_**ij**_ gives the probability of transition to the **low** risk state next season given outcome *i* for the farm 1 and outcome *j* for the farm 2. Similarly, ***M***_**ij**_ gives the probability of transition to the **medium** risk state next season given outcome *i* for the farm 1 and outcome *j* for farm 2. ***H***_**ij**_ gives the probability of transition to the **high** risk state next season given outcome *i* for the farm 1 and outcome *j* for the farm 2.

One option for specifying ***H, M*,** and ***L*** is as follows: We assume that if either farm experiences clinical infestation (i or *j* = 1), both farms transition to the high-risk state next year, represented as follows:

$$\begin{array}{c} H=\begin{array}{c} \begin{array}{cc} \begin{array}{cccc} 1 & 2 & 3 & 4 \end{array} & \; \end{array}\\ \begin{array}{cc} \left(\begin{array}{cccc} 1 & 1 & 1 & 1\\ 1 & 0 & 0 & 0\\ 1 & 0 & 0 & 0\\ 1 & 0 & 0 & 0 \end{array}\right) & \begin{array}{c} 1\\ 2\\ 3\\ 4 \end{array} \end{array} \end{array} \end{array}$$

We assume that if either farm correctly identifies subclinical infestation but neither clinical infestation (*i* = 2, *j* ≠ 1 or *j* = 2, *i* ≠ 1), both farms transition to the medium-risk state next year, represented as follows:

$$\begin{array}{l} M=\begin{array}{c} \begin{array}{cc} \begin{array}{cccc} 1 & 2 & 3 & 4 \end{array} & \; \end{array}\\ \begin{array}{cc} \left(\begin{array}{cccc} 0 & 0 & 0 & 0\\ 0 & 1 & 1 & 1\\ 0 & 1 & 0 & 0\\ 0 & 1 & 0 & 0 \end{array}\right) & \begin{array}{c} 1\\ 2\\ 3\\ 4 \end{array} \end{array} \end{array} \end{array}$$

If both farms are uninfested or they incorrectly identified subclinical infestation (*i* = 3 or 4, *j* = 3 or 4) both farms transition to the low risk state next year, represented as follows:

$$\begin{array}{c} L=\begin{array}{c} \begin{array}{cc} \begin{array}{cccc} 1 & 2 & 3 & 4 \end{array} & \; \end{array}\\ \begin{array}{cc} \left(\begin{array}{cccc} 0 & 0 & 0 & 0\\ 0 & 0 & 0 & 0\\ 0 & 0 & 1 & 1\\ 0 & 0 & 1 & 1 \end{array}\right) & \begin{array}{c} 1\\ 2\\ 3\\ 4 \end{array} \end{array} \end{array} \end{array}$$

Hence, the transition probabilities for the stochastic game are:

$$\begin{array}{l} H=\left(\begin{array}{cc} \begin{array}{cc} 1 & 1\\ 1 & 0 \end{array} & \begin{array}{cc} 1 & 1\\ 0 & 0 \end{array}\\ \begin{array}{cc} 1 & 0\\ 1 & 0 \end{array} & \begin{array}{cc} 0 & 0\\ 0 & 0 \end{array} \end{array}\right)\;M=\left(\begin{array}{cc} \begin{array}{cc} 0 & 0\\ 0 & 1 \end{array} & \begin{array}{cc} 0 & 0\\ 1 & 1 \end{array}\\ \begin{array}{cc} 0 & 1\\ 0 & 1 \end{array} & \begin{array}{cc} 0 & 0\\ 0 & 0 \end{array} \end{array}\right)\;L=\left(\begin{array}{cc} \begin{array}{cc} 0 & 0\\ 0 & 0 \end{array} & \begin{array}{cc} 0 & 0\\ 0 & 0 \end{array}\\ \begin{array}{cc} 0 & 0\\ 0 & 0 \end{array} & \begin{array}{cc} 1 & 1\\ 1 & 1 \end{array} \end{array}\right) \end{array}$$

As an example, consider a scenario in which neither farmer adopts the test. Suppose farm 1 had outcome *i* and farm 2 had outcome *j*, then the probability of this combination is DA_i_(θ_*H*_) × DA_j_(θ_*H*_). Therefore, the probability of these observations and followed by transitioning to the high-risk state next season would be

$$\begin{array}{l} \text{D}{\text{A}}_{\text{i}}\left(\theta_{H}\right)\text{ D}{\text{A}}_{\text{j}}\left(\theta_{H}\right)\;{\text{H}}_{\text{ij}} \end{array}$$

Thus, the overall probability of transition to the high-risk state will be obtained by summing over all *i* and *j*, i.e.,

$$\begin{array}{l} \overset{4}{\underset{i=1}{\sum }}\overset{4}{\underset{j=1}{\sum }}DA_{i}\left(\theta_{H}\right)DA_{j}\left(\theta_{H}\right)H_{ij}\; \end{array}$$

or, equivalently,

$$\begin{array}{l} \overset{4}{\underset{i=1}{\sum }}DA_{i}\left(\theta_{H}\right)\overset{4}{\underset{j=1}{\sum }}{H_{ij}DA}_{j}\left(\theta_{H}\right)\; \end{array}$$

Given that H_*ij*_ refers to the elements of a 4 × 4 matrix **H** and DA_*i*_(θ_*H*_) are the elements of a vector **DA**(θ_*H*_) of length 4, the sum over *i* and *j* is equal to multiplying the vector **DA**(θ_*H*_) by the matrix **H** and then multiplying the result by vector **DA**(θ_*H*_) i.e.,

$$\begin{array}{l} \text{D}\text{A}\left(\theta_{H}\right)\text{H}\cdot \text{D}\text{A}\left(\theta_{H}\right) \end{array}$$

Accordingly, for the complete stochastic game, the final payoff matrices and transition probabilities for the high risk state (blue), medium risk state (orange), and low risk state (green) are defined in Table 4. Note that the risk of being infected takes on θ_*K*_={θ_*L*_, θ_*M*_, θ_*H*_}, depending on the respective risk state of the farm last year, and **H** (high risk), **M** (medium risk), and **L** (low risk) represent the state-specific transition matrices.

**Table 4:** Matrix notation of the complete stochastic game.

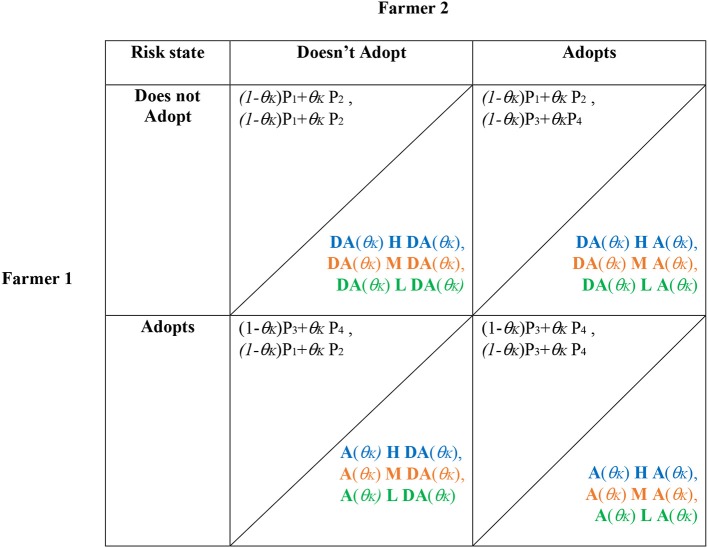

*For each strategy (adopts/does not adopt) chosen by each farmer (row values = Farmer 1; column values = Farmer 2), the upper triangle depicts the respective payoffs, whereas the lower triangle state the probabilities of transitioning to another risk state, shown in blue for transition to the high-risk state, orange for the medium-risk state and green for the low-risk state. Here, **H**, **M**, and **L** represent the transition matrix for each individual risk state (high, medium, low), which is multiplied by each farm's outcome vector of either adopting the ELISA test [A(θ_K_)] or not adopting the test [DA(θ_K_)]*.

## Results

We first examined the test adoption decision for each assumed risk state (*high, medium, low*) in terms of the Nash equilibrium and as a function of the extent to which farmers value future profits. This is captured in terms of the discount factor β. When β = 0, only immediate returns (i.e., within the season) factor into the farmers' decision and can be thought of as a short-term outlook; at the other extreme, when β = 1, all future returns are valued equally and can be thought of as taking a long-term outlook. At intermediate values of β, the further into the future a payoff comes, the less it is valued.

**Figure 4:**
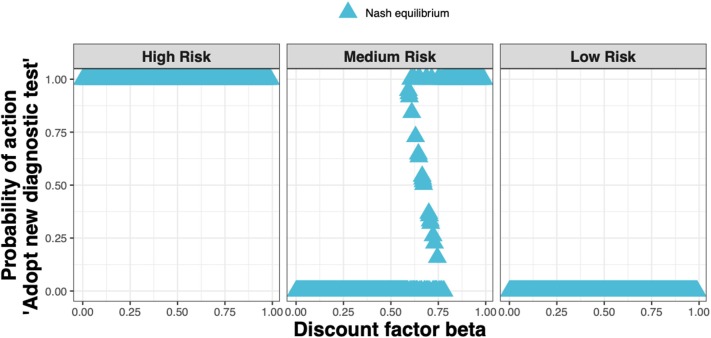
The Nash equilibrium probability in each of the three states (high, medium, and low risk) of adopting the new ELISA test when the cost is 1.5× the *status quo* clinical diagnosis cost, as a function of the discount factor.

When applying realistic economic and epidemiological parameters and an assumed cost for the new ELISA test of around 50% more than the *status quo* clinical diagnosis cost, we found that test adoption depends on the farmer's assumed infestation status (see Figure 4). Whenever a farmer considers their farm to be at high risk, i.e., if either farm had been diagnosed with clinical sheep scab the previous year, the diagnostic test will always be adopted. Whenever a farmer considers their farm to be at low risk, i.e., if neither farm had sheep scab the previous year, the test will never be adopted. However, when a farmer considers their farm to be at medium risk i.e., if either farm used the new ELISA test to diagnose and treat animals at the subclinical stage in the previous year, test adoption depends on the discount factor. In this case, mixed adoption can also be observed, meaning that the farmer will adopt the test with some probability between 0 and 1.

**Figure 5:**
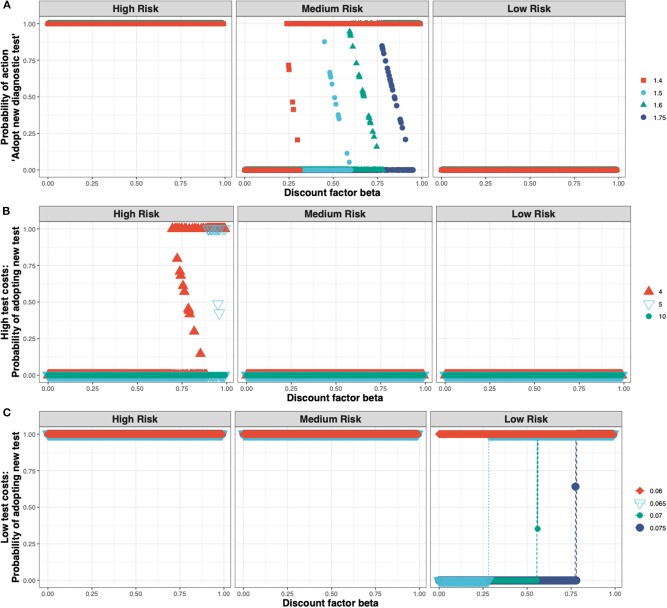
The Nash equilibrium probability in each of the three states (high, medium, and low risk) of adopting the new ELISA test as a function of the discount factor for different multiples of the status quo clinical diagnosis cost **(A)** medium test costs, shown for multiples of 1.4×, 1.5×, 1.6×, 1.75×; **(B)** high test costs, shown for multiples of 4×, 5×, 10×; and **(C)** low test costs, shown for multiples of 0.075×, 0.07×, 0.065×, 0.06×.

These outcomes are relatively robust toward the cost of the new ELISA test (see Figure 5A). The test costs would need to more than double before test adoption is not always observed in the high-risk state (see Figure 5B), and would need to be very low before test adoption can be seen in the low-risk state (see Figure 5C).

**Figure 6:**
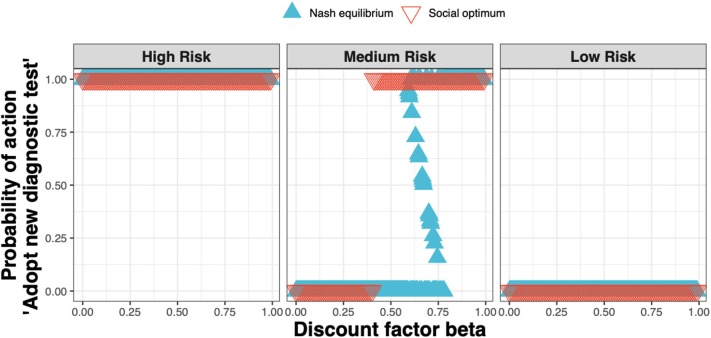
The Nash equilibrium (blue triangles) and the social optimum (red triangles) for a test cost 1.5× that of the *status quo* clinical diagnosis cost, as a function of the discount factor.

We found that the Nash equilibrium strategy does not always match the social optimum. For the same parameters as for Figure 4 (i.e., a new ELISA test cost 1.5× that of the *status quo* clinical diagnosis cost), in the high and low-risk states the strategies associated with the Nash equilibrium and social optimum agree; in the medium-risk state however, test adoption for the social optimum occurs at lower values of the discount factor than for the Nash equilibrium (Figure 6).

**Figure 7:**
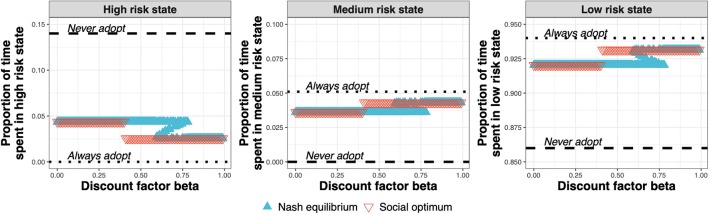
Proportion of time spent in the high-, medium-, and low-risk states for the Nash equilibrium (blue triangles), for the social optimum (red triangles), the status quo solution “*Never adopt”* and the solution “*Always adopt*” for test costs 1.5× that of the *status quo* clinical diagnosis cost, as a function of discount factor.

The test adoption decision determines the amount of time spent by the farms in the high-, medium-, and low-risk states (Figure 7). At the Nash equilibrium (Figure 7, blue triangles), compared to the status quo (*Never adopt*), less time is spent in the high-risk state, and more time spent in the medium and low-risk states. Compared to the Nash equilibrium, the social optimum (Figure 7, red triangles) either equals the Nash equilibrium or improves upon it by spending less time in the high-risk state and more time in the medium- and low-risk states.

**Figure 8:**
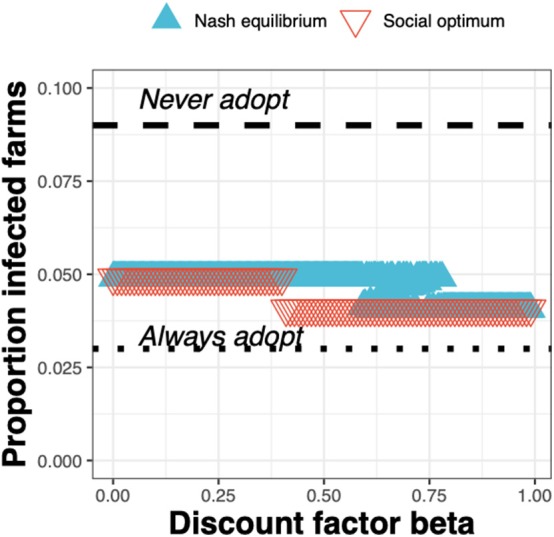
The proportion of infected farms at equilibrium for the Nash equilibrium (blue triangles), the social optimum (red triangles), the *status quo* “*Never adopt”* and the solution “*Always adopt”* for a test cost 1.5× than the *status quo* clinical diagnosis cost, as a function of the discount factor.

The epidemiological impacts can be observed in terms of the decrease in the proportion of infected farms when going from the *status quo* “*Never adopt”* (with a corresponding baseline proportion of infected farms of just under 0.1) to either the Nash equilibrium (Figure 8, blue triangles) or social optimum (Figure 8, red triangles), either of which result in a reduction in the proportion of infected farms of around a half.

**Figure 9:**
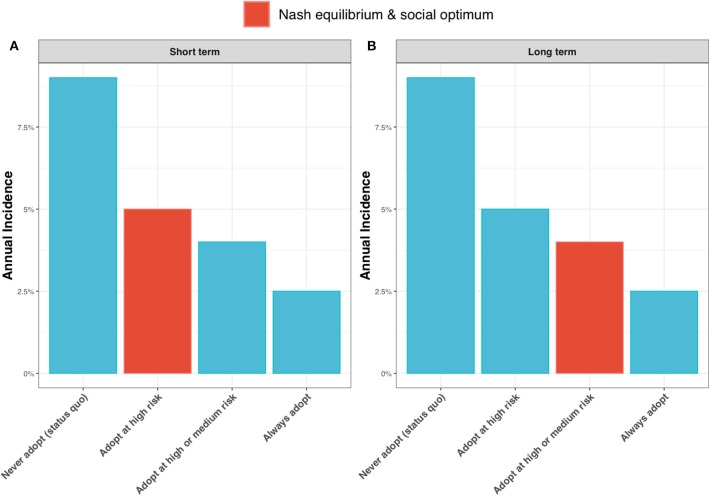
Annual incidence rate for **(A)** a short-term outlook (discount rate, β = 0) and **(B)** a long-term outlook (discount rate, β = 1) under alternative scenarios for adoption of the new ELISA test: the status quo (i.e., never adopting the test); always adopt the test (irrespective of risk state); adopt the test only in the high risk state; and adopt the test in the high and medium risk state. When β = 0, the Nash equilibrium and social optimum coincide (shown as red bar) and are to adopt the test in the high risk state (see Figure 6). When β = 1, the Nash equilibrium and social optimum coincide (shown as red bar) and are to adopt in the high and medium risk states (see Figure 6).

Contrasting the extreme cases of a discount factor of 0 (a short-term outlook) and a discount factor of 1 (a long-term outlook), shows the epidemiological outcome to be relatively robust to the discount factor. Under test adoption in the high-risk state only, the best strategy for a short-term outlook (Figure 9A, highlighted in red), the annual incidence rate is expected to drop to around 5%. If farmers take a long-term outlook and choose to also adopt the new ELISA test in the medium-risk state, the annual incidence rate is expected to drop further to 4% (Figure 9B, highlighted in red).

**Figure 10:**
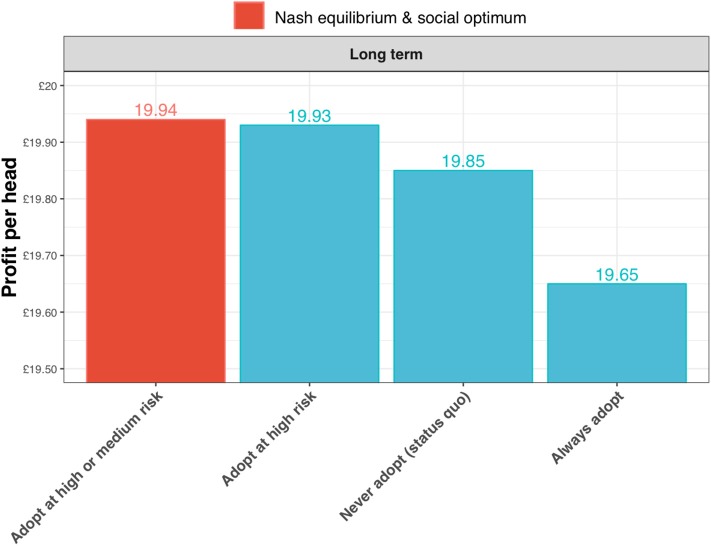
Expected profits per head for a long-term outlook (discount rate, β = 1) under alternative scenarios for adoption of the new ELISA test: the status quo (i.e., never adopting the test); always adopt the test (irrespective of risk state); adopt the test only in the high risk state; and adopt the test in the high and medium risk state. In this case of the long-term outlook, the Nash equilibrium and social optimum coincide at test adoption in the high and medium risk state (shown in red).

The expected profits per head are greatest for the strategy of adopting the test in the high and medium risk state (the Nash equilibrium and social optimum for the long-term outlook; Figure 10). The gains are relatively small, but nevertheless these results show that substantial reductions in annual incidence can be achieved without increasing costs to the farmer.

## Discussion and Future Work

The transmission and control of infectious diseases strongly depends on both the individual and joint decisions people make with regard to control measures and treatments. In this paper, we applied a game-theoretic model to examine the outcome of strategic interactions between neighboring farms, surrounding decisions to adopt a diagnostic test. The term *strategic interaction* is used because each farmer's decision and payoff depends on the decision made by their neighbor.

Specifically, the game-theoretic model applied in this paper was a stochastic game used to assess whether farmers are likely to adopt the new *P. ovis* diagnostic ELISA test for subclinical sheep scab and how this decision depends on whether a farmer considers their farm at high-, medium- or low-risk of infestation as well the costs and benefits of adopting the new test. Via the discount factor, a stochastic game also allows us to account for farmer preferences in terms of whether they take a short-term or long-term outlook and correspondingly whether they only factor immediate payoffs into their test adoption decision, or whether they factor in future benefits.

In Scotland, the *status quo* is that sheep scab diagnosis happens through skin scraping by a veterinary surgeon. The costs are assumed to come from just the veterinary surgeon call-out fee (here assumed to be £100 per flock), without a laboratory fee as this is currently paid for by the Scottish Government. The cost associated with using the new subclinical diagnostic test is assumed to be the vet call out fee plus laboratory costs (here assumed to be £60 per flock, assuming 12 animals are tested at £5 each). Thus, our assumptions here are that using the new ELISA test would come to around £160 per flock vs. the £100 per flock for the *status quo* clinical diagnostic test.

We analyzed the test adoption outcomes (*Nash equilibria*) and showed that that they are strongly-dependent on the assumed risk state (*high, medium, low*), and also that they are modulated not just by the costs of the new diagnostic test but also by how much short-term profits are preferred over long-term benefits. When applying realistic economic and epidemiological parameters and using an expected test cost of around 50% more than the current clinical diagnosis via skin scraping, we observed test adoption in the high-risk state, no test adoption in the low-risk state, and mixed strategies in the medium risk state that depended on the preference for short-term over long-term profits. We found the outcomes in terms of test adoption to be relatively robust to the cost of the test, with substantial increases or decreases in test cost required to change the overall pattern of test adoption.

Individual decisions in game-theoretic models are based on assumptions of rational self-interest and do not necessarily correspond to a socially optimal outcome. However, in our analysis, we found that test adoption decisions at the Nash equilibrium were socially optimal for most calculated outcomes. Specifically, whenever a farmer considered their farm to be at high risk based on last year's infestation status of themselves or their neighbor's they always chose to adopt the new diagnostic test. Analogously, whenever a farmer considered their farm to be at low risk given that neither farm had sheep scab the previous year, the test was never adopted. For our default parameters—an expected test cost of around 50% more than the current clinical diagnosis via skin scraping—we found discrepancies between the Nash equilibrium and the social optimum in the medium risk state, for intermediate values of the discount factor (the parameter specifying the preference for short-term over long-term gains). One reason for this is that adopting the new diagnostic test is freely chosen by the individual farmers and individual choices do not necessarily align with the public interest. Also, some individuals may free-ride on the protection provided by their neighbor, which is at odds with the socially optimal outcome. In light of this any new policy intervention promoting the use of the new diagnostic ELISA must address the divergence between private and public consequences of actions and, ideally, motivate individual free choice toward a social optimum (28). This could be achieved for example by offering private incentives and encouraging cooperative schemes among farmers.

However, when viewing the outcomes in terms of the prevalence of infestation, we see that the outcomes are largely robust to whether the Nash equilibrium or social optimum is adopted. The primary benefits are seen in the drop in prevalence from the baseline of just under 10% to around 5% following adoption of the test in the high-risk state, or 4% following adoption in the high- and medium risk state.

The financial benefits to the farmer are not substantial; however, what these results show is that substantial reductions in sheep scab incidence should be achievable without additional costs to the farming community. Moreover, the results suggest that the primary goal should be to facilitate test adoption amongst farms at high-risk of infestation, as this would provide most of the epidemiological benefits.

However, there are limitations to this modeling framework. Firstly, the framework does not account for the fact that the external risk to farms should decline as the expected prevalence of infestation in the farms adopting the test declines. Whilst capturing this would be desirable, it is not something that can readily be done within the stochastic game framework. We therefore view our results as a conservative assessment of the benefits of the adopting the test, since widespread adoption would reduce the external risk to farms. Thus, cooperative behavior among the farming community should provide additional benefits, first by encouraging the social optimum rather than just the Nash equilibrium outcome, and secondly, by reducing the external risk of infestation and therefore the expected prevalence of infestation following widespread test adoption.

A second limitation is that the model considers a two-farm system only and the above scenario of widespread adoption should ideally be assessed by extending the analysis to include multiple farms as well as multiple farmer strategies. In this paper we chose to examine this simple two-farm set-up in order to allow a game-theoretic approach. However, to capture multiple farms with realistic farm to farm heterogeneity and features such as explicit import of infested sheep (as opposed to capturing this via the fixed background risk) would require a much more flexible framework. Such future work would be more amenable to an agent-based approach, following, for example (29). This could offer a more flexible and potentially more realistic approach for modeling individual farmer decisions that allows farmers to learn from their infestation history, risk status, and past payoffs. The approach in this paper necessarily assumes a perfect mixing of populations, and is not able to capture farm heterogeneity and differences in behavior, not only socially but also in terms of spatially-driven interactions. Particularly for the latter, where players interact with their immediate neighbors more than with randomly chosen individuals and the payoff becomes a function of the risk state and preferred choices of more than two players, agent-based methods on a network or a grid-based scenario provide a potential next step.

Third, the costs used in these analyses were current at the time of publication of the ADAS report and some changes may have occurred. Nevertheless, the robustness of the results to test cost would suggest that we would expect a similar picture with current figures.

It should also be noted that the results shown here assume a test with perfect sensitivity and specificity—a reasonable assumption given that estimates for flock level sensitivity and specificity are very high at 0.98 and 0.97, respectively. However, the framework can be used in the case of an imperfect test (as described in the Supplementary Material). When we assume an imperfect test, we obtain broadly similar results, albeit with slightly lower payoffs and slightly higher incidences of infestation. Small improvements in outcome were observed for a scenario in which the farmer *does not know* that the test is imperfect, vs. a scenario whereby the farmer is assumed to be able to captured changes to payoffs due to an imperfect test in the calculation of the Nash equilibrium.

In summary, we have presented a novel use of a stochastic game which provides advantages over the more commonly used static games [e.g., (21)], especially in an epidemiological context where it is useful to be able to capture dynamic changes in risk. Together our findings provide strong support for the new diagnostic test whilst also indicating that further benefits could be accrued through flock health schemes that encourage and facilitate cooperation between farmers. Our key finding however, remains that adopting the new diagnostic ELISA test for subclinical sheep scab could significantly reduce prevalence of sheep scab and improve animal welfare in a cost-neutral way to the industry.

## Data Availability Statement

The datasets generated for this study are available on request to the corresponding author.

## Author Contributions

SM wrote the manuscript, ran simulations, conducted analysis, and interpreted the results. RB, AN, SB, RR, MD, TP, SM, RZ, and LM developed the methodology, performed analysis, and contributed to the manuscript. RR and MD provided R code for the analyses. All authors reviewed the final manuscript.

### Conflict of Interest

The authors declare that the research was conducted in the absence of any commercial or financial relationships that could be construed as a potential conflict of interest.

## Footnotes

^1^A farm is at a medium risk of infestation if sheep scab was diagnosed using the new test and then treated at the subclinical stage.
