# Supplementary material for: Uptake of Diagnostic Tests by Livestock Farmers: A Stochastic Game Theory Approach
Source: Front Vet Sci. 2020 Feb 5;7:36. doi: 10.3389/fvets.2020.00036 (PMC7012806; doi:10.3389/fvets.2020.00036)
Supplement: Supplementary file 1 [file Data_Sheet_1.pdf]

Appendix

A stochastic game is defined in terms of *payoff* and *transition probability matrices* in each of *n* states. For 2 players and 2 actions the payoffs and transition probabilities are expressed as follows:

$$R^k(s) = [r^k(s, a^1, a^2)]_{a^1=1,\, a^2=1}^{2,2} \qquad \text{(4a)}$$

which is shorthand for

$$R^k(s) = \begin{bmatrix} r^k(s, a^1 = 1, a^2 = 1), & r^k(s, a^1 = 1, a^2 = 2) \\ r^k(s, a^1 = 2, a^2 = 1), & r^k(s, a^1 = 2, a^2 = 2) \end{bmatrix} \qquad \text{(4b)}$$

Thus, $R^k(s)$ is the *k*-th player's immediate reward matrix in state *s,* given action a^1 by player 1 and action a^2 by player 2. The actions a^1 and a^2 take values 1 or 2 to indicate whether the player plays the first or second action. The expression $p(s'|s, a^1, a^2)$ gives the probability of going from state *s* to s' given action a^1 by player 1 and action a^2 by player 2. The actions played by each player in each state are expressed mathematically in terms of the strategy vector $\underline{\boldsymbol{f}}$ for player 1 and and $\underline{\boldsymbol{g}}$ for player 2. It is convenient to write $\underline{\boldsymbol{f}}(s)$ as a row vector $\underline{\boldsymbol{f}}(s) = \big(f(s, a^1 = 1), f(s, a^1 = 2)\big)$ with elements giving the probability of each action in state s, and $\underline{\boldsymbol{g}}(s)$ as a column vector

$$\underline{\boldsymbol{g}}(s) = \begin{pmatrix} g(s, a^2 = 1) \\ g(s, a^2 = 2) \end{pmatrix} \qquad \text{(5)}$$

with elements giving the probability of each action in state *s*.

1.1.1 Transition matrix and dynamic behavior.

The strategy vectors $\underline{\boldsymbol{f}}$ and $\underline{\boldsymbol{g}}$ determine the dynamics of the system in terms of the stochastic transitions between states as follows: given these strategies the probability $p\left(s' \middle| s, \underline{\boldsymbol{f}}, \underline{\boldsymbol{g}}\right)$ of transitioning from state *s* to state s' is given by the sum

$$p\left(s' \middle| s, \underline{\boldsymbol{f}}, \underline{\boldsymbol{g}}\right) = \sum_{a^1=1,\, a^2=1}^{2,2} p(s'|s, a^1, a^2) f(s, a^1) g(s, a^2) \qquad \text{(6a)}$$

where $f(s, a^1)$ is the probability of action a^1 being played by player 1 in state *s* and $g(s, a^2)$ is the probability of action a^2 being played by player 2 in state *s*. This expression gives one element of an *n* by *n* stochastic transition matrix $P(\underline{\boldsymbol{f}}, \underline{\boldsymbol{g}})$ for which the element in row *s* and column s' is the probability of transitioning from state *s* to state s', i.e.

$$P(\underline{\boldsymbol{f}}, \underline{\boldsymbol{g}}) = \left[p\left(s' \middle| s, \underline{\boldsymbol{f}}, \underline{\boldsymbol{g}}\right)\right]_{s=1, s'=1}^{n,n} \qquad \text{(6b)}$$

with *n* the number of states. To summarise, for any strategy vectors $\underline{\boldsymbol{f}}$ and $\underline{\boldsymbol{g}}$ we can write down $P(\underline{\boldsymbol{f}}, \underline{\boldsymbol{g}})$ which determines the stochastic transitions between states. More precisely, $P(\underline{\boldsymbol{f}}, \underline{\boldsymbol{g}})$ defines a *Markov chain*. A Markov chain represents any stochastic transition process between states where the

probability of transition depends on the current state and not the history of states visited. As there are payoffs or rewards associated with the states visited by the Markov chain, this represents a *Markov reward process.*

1.1.2 The beta discounted reward.

There is one additional quantity needed before the stochastic game is complete and the Markov reward process can be defined further. This is the value of the *discounted reward* parameter (β), here also referred to as *discount factor*, which can take on values between 0 and 1. This parameter determines how much the farmer values his future payoffs over his current payoffs. Mathematically, the value of β specifies the weight given to next year's payoff relative to the current payoff. Taking the extreme cases, if β=0, next year's payoff carries no weight in the decision making. If β=1, next year's payoff carries equal weight to the current payoff. More general, any monetary reward that might be earned in the future is less valuable than the same monetary reward earned today. As an example of its derivation, consider an interest rate x. An interest rate x means than an amount of money M today would be worth $M(1+x)$ in one year's time and $M(1+x)^n$ in n years time. Conversely, an amount M_k earned k years in the future is worth of $M_k /(1+x)^k$ today. Setting

$$\beta = \frac{1}{(1+x)} \qquad (7)$$

then the current worth of an amount M_k earned k years in the future can be written $M_k \, \beta^{k}$. The total worth of current and future payoffs is given by the discounted sum

$$M_0 + M_1\beta + M_2\beta^2 + M_3\beta^3 + \cdots + M_k\beta^k + \cdots$$

The weights $1, \beta, \beta^2, \beta^3, \ldots, \beta^k, \ldots$ sum to $1/(1-\beta)$. Therefore, we can obtain a normalised sum by scaling the weights by $(1-\beta)$. This gives the standard **beta discounted sum**

$$D_\beta(M_0, M_1, M_2 \ldots) = (1-\beta)M_0 + (1-\beta)(M_1\beta + M_2\beta^2 + M_3\beta^3 \ldots) \qquad (8)$$

This is connected to the Markov reward process (31) such that if there is a Markov chain determining the visits to future states, and therefore the series of future payoffs, then future rewards will need to be discounted.

1.1.3 The Markov reward process.

Consider the example of a simple system with only 1 state. Define v^k to be the long-term value accrued by player k and let r^k be the payoff to player k at each time. Then

$$\mathrm{v}^k = r^k + \beta r^k + \beta^2 r^k + \beta^3 r^k + \cdots$$

which can be rewritten as

$$\mathrm{v}^k = r^k + \beta(r^k + \beta r^k + \beta^2 r^k + \cdots)$$

i.e.

$$\mathrm{v}^k = r^k + \beta \mathrm{v}^k \qquad (9)$$

which can be rearranged to give $\mathrm{v}^k = \frac{r^k}{(1-\beta)}$. Now consider the general case with multiple states and let $\mathrm{v}^k(s)$ be the long term value accrued by player k starting in state *s*. Assuming the process starts at time *t*, the long term value, $\mathrm{v}^k(s)$, must equal the payoff at time *t* when in state *s*, given by $r^k\left(\mathrm{s}, \underline{\boldsymbol{f}}, \underline{\boldsymbol{g}}\right)$, plus β multiplied with the expected long-term value accrued from the state S_{t+1} given that the state at time *t* was *s*, i.e.

$$\mathrm{v}^k(s) = r^k\left(\mathrm{s}, \underline{\boldsymbol{f}}, \underline{\boldsymbol{g}}\right) + \beta\mathbb{E}\left(\mathrm{v}^k(S_{t+1}|S_t = s)\right) \qquad (10)$$

The expected value $\mathbb{E}$ of $\mathrm{v}^k(S_{t+1}|S_t = s)$ is derived by weighting the value in state s' by the probability of transitioning to that state *i.e.*

$$\mathbb{E}(\mathrm{v}^k(S_{t+1}|S_t = s)) = \sum_{s'} P(\underline{\boldsymbol{f}}, \underline{\boldsymbol{g}})_{ss'}\, \mathrm{v}^k(s')$$

Let $\underline{\mathrm{v}}^k$ be the vector (of length equal the number of states) of long-term values for player *k*, then in matrix form, equation (10) is written

$$\underline{\mathrm{v}}^k = \underline{r}^k\left(\underline{\boldsymbol{f}}, \underline{\boldsymbol{g}}\right) + \beta\, P(\underline{\boldsymbol{f}}, \underline{\boldsymbol{g}})\, \underline{\mathrm{v}}^k \qquad (11)$$

To find the Nash equilibrium, for any $\underline{\boldsymbol{f}}$ and $\underline{\boldsymbol{g}}$, equation (11) is solved for $\underline{\mathrm{v}}^k$ to give

$$(I - \beta\, P(\underline{\boldsymbol{f}}, \underline{\boldsymbol{g}}))\, \underline{\mathrm{v}}^k = \underline{r}^k\left(\underline{\boldsymbol{f}}, \underline{\boldsymbol{g}}\right)$$

where *I* is the identity matrix, and thus $\underline{\mathrm{v}}^k = (I - \beta\, P(\underline{\boldsymbol{f}}, \underline{\boldsymbol{g}}))^{-1}\ \underline{r}^k\left(\underline{\boldsymbol{f}}, \underline{\boldsymbol{g}}\right)$. **For instance, consider the payoffs returned to each player under a fixed strategy played by the one player:** The matrix $T(s, \mathrm{v}^k)$ above defines the elements of which the expected long-term value accrued after starting in state *s* and following transition to another state determined by actions a^1 and a^2.

$$T\left(s, \underline{\mathbf{v}}\right) = \left[\sum_{s' \in S} p(s'|s, a^1, a^2)\mathrm{v}(s')\right]_{a^1=1,\, a^2=1}^{2,2}$$

If player 2 plays strategy $\underline{\boldsymbol{g}}(s)$, then player 1 receives a current payoff given by the vector

$$R^1(s)\underline{\boldsymbol{g}}(s)$$

where the elements give the payoff for each of the possible actions taken by player 1. If we assume that player 1 knows how to play optimally from the next time point then the expected future payoff for each of the actions player 1 might take is given by the elements of the vector

$$T\left(s, \underline{\mathrm{v}}^1\right)\underline{\boldsymbol{g}}(s)$$

Therefore, the total discounted future reward from playing each of the possible actions is given by the elements of the vector

$$R^1(s)\underline{\boldsymbol{g}}(s) + \beta T\left(s, \underline{\mathrm{v}}^1\right)\underline{\boldsymbol{g}}(s) \qquad (12)$$

Correspondingly, if player 1 plays strategy $\underline{\boldsymbol{f}}(s)$, then player 2 receives a current payoff given by the vector

$$\underline{\boldsymbol{f}}(s)R^2(s)$$

and assuming that player 2 knows how to optimally play from the next time point, then the expected future payoff for each of the actions player 2 might take is given by the elements of the vector

$$\underline{\boldsymbol{f}}(s)T\left(s, \underline{\mathrm{v}}^2\right)$$

Therefore, the total discounted future reward from playing each of the possible actions is given by the elements of the vector

$$\underline{\boldsymbol{f}}(s)R^2(s) + \beta\underline{\boldsymbol{f}}(s)T\left(s, \underline{\mathrm{v}}^2\right) \qquad (13)$$

1.2 The Nash equilibrium and the pareto and social optimum.

The final step is to specify the constraints on $\underline{\boldsymbol{f}}$ and $\underline{\boldsymbol{g}}$ that ensure that the outcome is a Nash equilibrium. The idea is to view the matrices given in expressions (3) and (4) as straightforward payoff matrices in a one-stage game as in the initial example. Thus, expression (3) is equivalent to matrix A (see section'Basic definitions' of the main manuscript) and expression (4) is equivalent to matrix B, and $\underline{\boldsymbol{f}}$ and $\underline{\boldsymbol{g}}$ are equivalent to X and Y. The only difference is that the simple game had one state, whereas we now have constraints for each state. The appropriate constraints are therefore given by

$$R^1(s)\underline{\boldsymbol{g}}(s) + \beta T\left(s, \underline{\mathrm{v}}^1\right)\underline{\boldsymbol{g}}(s) \leq \mathrm{v}^1(s)\mathbf{1}_2$$

$$\underline{\boldsymbol{f}}(s)R^2(s) + \beta\underline{\boldsymbol{f}}(s)T\left(s, \underline{\mathrm{v}}^2\right) \leq \mathrm{v}^2(s)\mathbf{1}_2^{\boldsymbol{T}}$$

where $\mathbf{1}_2 \begin{pmatrix} 1 \\ 0 \end{pmatrix}$ and $\mathbf{1}_2^{\boldsymbol{T}}$ (1, 1) and $\mathrm{v}^1(s)$ is the value to player 1 in state *s* when playing the Nash equilibrium strategy $\underline{\boldsymbol{f}(s)}$, and $\mathrm{v}^2(s)$ is the value to player 2 in state *s* when playing the Nash equilibrium strategy $\underline{\boldsymbol{g}(s).}$ Nash equilibria are not necessarily Pareto-optimal. The Pareto-optimal solution is a set of strategies in which no player's expected gain can be improved upon without decreasing the expected gain of any other player. A game may have many Pareto optima, but in this paper we investigate the Pareto optimum which is also a *social optimum*. The *social optimum* is defined as the solution for which the sum of payoffs is maximised. Note that a social optimum must also be a Pareto optimum because if it were possible to find a strategy that improves the payoff of one player without reducing the payoff of another then this implies a potential social optimum instead.

Numerical solution

To solve the stochastic game we apply nonlinear optimization routines using the R software package version 3.4.2 (32), minimizing the objective function

$$\sum_{k=1,2} \mathrm{I}^T \left[\underline{\mathrm{v}}^k - \underline{r}^k\left(\underline{\boldsymbol{f}}, \underline{\boldsymbol{g}}\right) - \beta \mathrm{P}\left(\underline{\boldsymbol{f}}, \underline{\boldsymbol{g}}\right) \underline{\mathrm{v}}^k\right] \qquad (14)$$

where k refers to the number of the respective player, I is the identity matrix, $\underline{\mathrm{v}}^k$ is the value vector for player k, $\left(\underline{\boldsymbol{f}}, \underline{\boldsymbol{g}}\right)$ represents the vector of expected payoffs, and β denotes the discount factor, which is then multiplied with the stochastic transition matrix $\mathrm{P}\left(\underline{\boldsymbol{f}}, \underline{\boldsymbol{g}}\right) \underline{\mathrm{v}}^k$.
