# Supplementary material for: Uptake of Diagnostic Tests by Livestock Farmers: A Stochastic Game Theory Approach
Source: Front Vet Sci. 2020 Feb 5;7:36. doi: 10.3389/fvets.2020.00036 (PMC7012806; doi:10.3389/fvets.2020.00036)
Supplement: Supplementary file 2 [file Data_Sheet_2.pdf]

Supplementary Material 1: Estimation of probabilities of infestation

The probabilities of infestation in the high risk state (θ_H) and in the low risk state (θ_L) were estimated using data from a cross-sectional study of sheep farms asked whether they had sheep scab outbreaks in the study year and how many outbreaks they had experienced in the previous 10 years (5). Of the 383 farms for which valid questionnaires were returned, 33 reported at least one outbreak in the current year (just under 9%), of which 19 had experienced 1-5 outbreaks and 9 had experienced outbreaks > 5 of the previous 10 years. Of the 266 farms which reported no current outbreak, only 84 experienced an outbreak in the previous 10 years.

Sheep scab transmission was simulated using a stochastic two-farm metapopulation model, where infestation can occur from either bought in sheep or through local transmission from a neighbouring flock. Specifically, the parameters θ_H and θ_L were used to describe a Markov chain with states describing the joint infestation status of the two farms, namely “Both infested”, “One infested” or “Neither infested”.

The Markov chain can be described by the transition matrix M, where

$$M = \begin{pmatrix} \theta_H^2 & 2\theta_H(1-\theta_H) & (1-\theta_H)^2 \\ \theta_H^2 & 2\theta_H(1-\theta_H) & (1-\theta_H)^2 \\ \theta_L^2 & 2\theta_L(1-\theta_L) & (1-\theta_L)^2 \end{pmatrix}$$

and the element M_{ij} gives the probability of transitioning from state i to state j. We assume that if at least one of the farms is infested (“Both infested” or “One infested) the probability of either farm being infested next time is θ_H. If neither farm is infested the probability of being infested next time is θ_L.

The Markov chain was used to simulate an 11 year infestation history (to match the observed data) 10000 times. At the end of the 11 year infestation history, a state of “Both infested” meant that we recorded an outbreak; “Neither infested” meant we recorded no outbreak; and “One infested” meant we recorded an outbreak with a 0.5 probability. To correspond with the available data, for cases where an outbreak was recorded, we also recorded whether 1-5 or > 5 outbreaks had occurred in the previous 10 years; and for cases where no outbreak was recorded, we also recorded whether some or no outbreaks had occurred in the previous 10 years. This allowed us to obtain the probability of the observed data for any pair θ_H and θ_L and identify the maximum likelihood estimates for these parameters.

Supplementary Material 2: Multistate model setup with imperfect test

1 Background

The performance of a diagnostic test is defined by two concepts, i.e. test *sensitivity* (*Se*) and test *specificity* (*Sp*). Sensitivity is the probability of correctly diagnosing infestation if it is present, whereas specificity is 1- the probability of incorrectly diagnosing infestation when it is absent. Here, we can

define group level sensitivity and specificity for the ELISA blood test, applied to *n* number of animals. The probability of detection in a flock is then

$$P_{Detect} = 1\text{-}(1\text{-}Se)^{n}$$

The probability of obtaining a false positive in the group / flock is

$$P_{False.Positive} = 1\text{-}(Sp)^{n}$$

For the imperfect test, we assume test sensitivity *Se*=0.98 and test specificity *Sp*=0.97 (11).

2 Payoffs.

First consider the payoffs a farmer receives under 4 scenarios (with the red font indicating where these expressions differ from those in the main text shown for a perfect test):

① The farmer's flock is **uninfested** (although he doesn't know this) and he **does not** adopt the new blood test

- $P_1 = R_H$

② The farmer's flock is **infested** (although he doesn't know this) and he **does not** adopt the new blood test

- $P_2 = (1 - \varphi_1)\, R_H + (\varphi_1 - \varphi_2)\, R_S + \varphi_2 R_C - (C_{Diag}+C_{Treat})$

③ The farmer's flock is **uninfested** (although he doesn't know this) and he **does** adopt the new blood test

- $P_3 = R_H - C_{Test} - P_{False.Positive}\, C_{Treat}$

④ The farmer's flock is **infested** (although he doesn't know this) and he **does** adopt the new blood test

- $P_4 = P_{Detect}[(1 - \varphi_1)\, R_H + \varphi_1\, R_{TT} - (C_{Test}+C_{Treat})] + (1\text{-}P_{Detect})[(1 - \varphi_1)\, R_H + (\varphi_1 - \varphi_2)\, R_S + \varphi_2 R_C - (C_{Test}+C_{Diag}+C_{Treat})\,]$

Variable	Description
R_H	Revenue from healthy animals
R_S	Revenue from subclinically infested animals
R_C	Revenue from clinically animals
R_{TT}	Revenue from tested and treated animals
φ_1	Total prevalence in an infested flock
φ_2	Clinical prevalence in an infested flock
C_{Diag}	Cost of clinical diagnosis
C_{Treat}	Cost of treatment
C_{Test}	Cost of new diagnostic test

Table 1.: Assumed and derived parameters.

3 Outcome probabilities.

To decide whether the farmers will consider themselves in a high, medium, or low risk state the following year using an imperfect test setting, we need to consider what the possible outcomes are this season, dependent on their actions. The four possible outcomes for a given flock at the end of the season are

1) clinical infestation is observed and treated
2) subclinical infestation is correctly identified (although the farmer does not know this) and treated
3) subclinical infestation is incorrectly identified (although the farmer does not know this) and treated
4) no infestation is correctly identified (although the farmer does not know this) and treatment is not administered.

Outcome 1 (clinical infestation is observed and treated) would happen if a flock was infested and progressed to clinical infestation but the farmer had not tested the flock, or tested and received false negatives. Outcome 2 (subclinical infestation is correctly identified and treated) would happen if the flock was infested and tested positive. Outcome 3 (subclinical infestation is incorrectly identified) would happen if the flock was uninfested and testing returns false positives. Outcome 4 (no infestation is diagnosed) would happen if the flock is uninfested and testing gives no false positives.

The vector of outcome probabilities (see Supplementary Table 2) for adopting the test $A(\theta)$ and not adopting the test $DA(\theta)$ using an imperfect test are

$$A(\theta) = \begin{pmatrix} (1 - P_{Detect})\,\theta \\ P_{Detect}\,\theta \\ P_{False.Positive}\,(1-\theta) \\ (1 - P_{False.Positive})\,(1-\theta) \end{pmatrix} \text{ and } DA(\theta) = \begin{pmatrix} \theta \\ 0 \\ 0 \\ 1-\theta \end{pmatrix}$$

Outcome probabilities when adopting or not adopting the test	Outcome probabilities expressed in terms of model parameters
$A_1(\theta)$	$(1-P_{Detect})\ \theta$
$A_2(\theta)$	$P_{Detect}\ \theta$
$A_3(\theta)$	$P_{False.Positive}\ (1-\theta)$
$A_4(\theta)$	$(1-P_{False.Positive})(1-\theta)$
$DA_1(\theta)$	θ
$DA_2(\theta)$	0
$DA_3(\theta)$	0
$DA_4(\theta)$	$(1-\theta)$

Supplementary Table 2. Outcome probabilities expressed in terms of the epidemiological parameters in Table 2 of main manuscript.

4 Transition probabilities and final pay-off matrices

Here, we present adjusted transition probabilities to account for the transitions the farmer *believes* will occur. For example, if the test is used and a false positive is obtained (outcome 3) on either farm, each farmer will believe his farm to be in the medium risk state the following year, whereas the actual transition would be to the low risk state if the true state of the farms is uninfested.

Transition probabilities associated with the farmer beliefs of moving between high, low, and medium risk states are defined as follows: Analogous to the perfect test case **H, M** and **L** are equal 4x4 matrices, which satisfy $L_{ij} + M_{ij} + H_{ij} = 1$, and the four possible belief outcomes for a given flock at the end of the season are (i) clinical infestation is observed and treated, (ii), subclinical infestation is *correctly* identified and treated, (iii) subclinical infestation is *incorrectly* identified and treated, and (iv) the absence of infestation is *correctly* identified.

If either farm experiences clinical infestation, both farms transition to the high-risk state next year (the column values refer to the outcomes (1-4) for farm 1 and the row values refer to the outcomes (1-4) for farm 2), *i.e.*

$$\begin{matrix} & \begin{matrix} 1 & 2 & 3 & 4 \end{matrix} & \\ \boldsymbol{H} = & \begin{pmatrix} 1 & 1 & 1 & 1 \\ 1 & 0 & 0 & 0 \\ 1 & 0 & 0 & 0 \\ 1 & 0 & 0 & 0 \end{pmatrix} & \begin{matrix} 1 \\ 2 \\ 3 \\ 4 \end{matrix} \end{matrix}$$

In the transition matrices below, the red entries indicate where the transitions based on farmer beliefs differ from the actual transitions.

If either farm believes they have seen subclinical infestation, but no clinical infestation, they believe they transition to the medium risk state, i.e.

$$\begin{matrix} & \begin{matrix} 1 & 2 & 3 & 4 \end{matrix} & \\ \boldsymbol{M} = & \begin{pmatrix} 0 & 0 & 0 & 0 \\ 0 & 1 & 1 & 1 \\ 0 & 1 & 1 & 1 \\ 0 & 1 & 1 & 0 \end{pmatrix} & \begin{matrix} 1 \\ 2 \\ 3 \\ 4 \end{matrix} \end{matrix}$$

If both farms believe no infestation was present, they believe they will transition to the low risk state, i.e.

$$\begin{matrix} & \begin{matrix} 1 & 2 & 3 & 4 \end{matrix} & \\ \boldsymbol{L} = & \begin{pmatrix} 0 & 0 & 0 & 0 \\ 0 & 0 & 0 & 0 \\ 0 & 0 & 0 & 0 \\ 0 & 0 & 0 & 1 \end{pmatrix} & \begin{matrix} 1 \\ 2 \\ 3 \\ 4 \end{matrix} \end{matrix}$$

The final pay-off matrices remain the same as specified in section **4.3** of the main manuscript.

4.1 Nash equilibrium & social optimum

In contrast to the multistate setup for the perfect test described in the main manuscript, for the imperfect test there are clear distinctions between the payoffs and transition probabilities used by the farmer for calculating the Nash equilibrium and the social optimum.

We consider two possible imperfect test scenarios: (i) Farmers are aware of the adjusted payoffs due to imperfect test and (ii) Farmers believe the test to be perfect and are not aware of adjusted payoffs. In both cases, farmers are assumed to operate on the basis of their believed transitions between the risk states. The social optimum was calculated using the adjusted payoffs together with the true transitions between states.

For the case of the farmers valuing long-term gains (discount rate, $\beta = 1$), we compared the outcome (Nash equilibrium and social optimum) of the perfect test with those of the imperfect test scenarios. In both cases, we found that the annual incidence at the Nash equilibrium and social optimum was slightly higher for the imperfect test than for the perfect test (Fig. S1 and Fig. S2). We also observed that when the farmer was aware of the adjusted payoffs (case (i)), the Nash equilibrium differed from the social optimum (Fig. S1, left hand side), but when the farmer is unaware of the adjusted payoffs, the Nash equilibrium and social optimum coincide (Fig. S1, right hand side). Thus, when farmers are not aware of the imperfect test sensitivity and specificity, a slightly improved outcome is obtained.

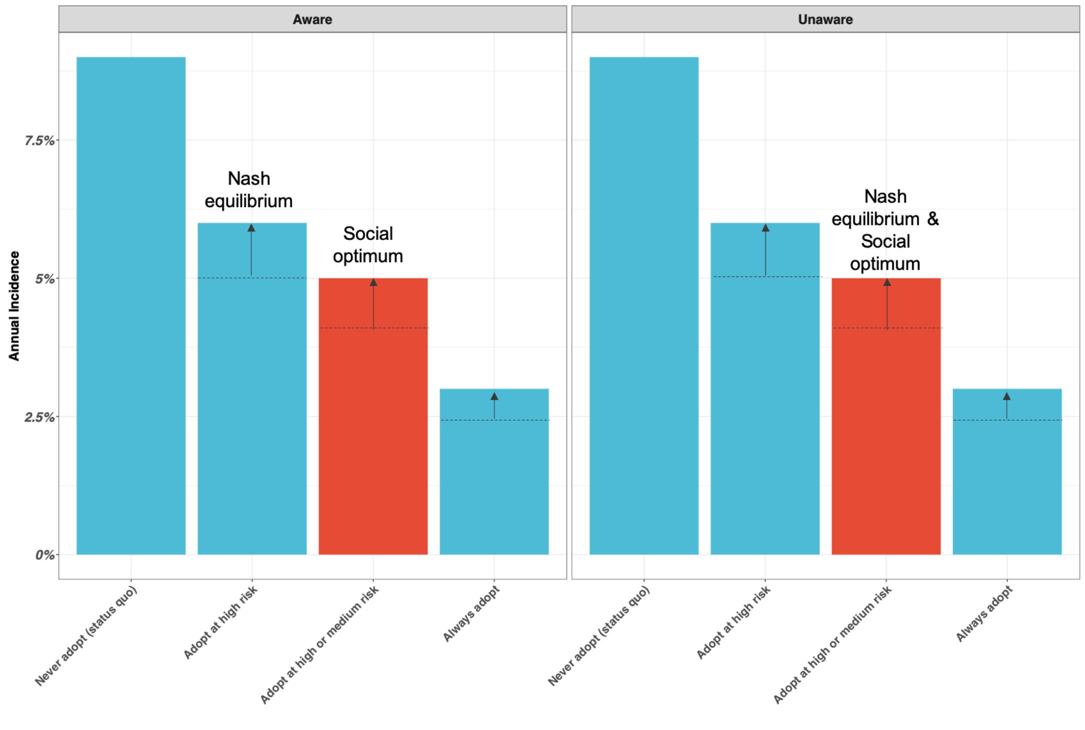

Figure S1. Left: Best strategy for imperfect test scenario (Se=0.98, Sp=0.97) when farmers are **aware** of the adjusted payoffs. The dashed horizontal line indicates the outcome for the perfect test scenario (Se=1, Sp=1). **Right:** Best strategy for imperfect test scenario (Se=0.98, Sp=0.97) when farmers are **unaware** of the adjusted payoffs. The dashed line indicates the outcome for the perfect test scenario (Se=1, Sp=1).
